# 3D finite element analysis of stress distribution as a result of oblique and horizontal forces after regenerative endodontic treatment part II: comparison of material thickness

**DOI:** 10.1186/s12903-023-03559-x

**Published:** 2023-11-16

**Authors:** Beril Demircan, Pınar Demir

**Affiliations:** Department of Pediatric Dentistry, Faculty of Dentistry, Nuh Naci Yazgan University, Kayseri, 38170 Kocasinan Türkiye

**Keywords:** Regenerative endodontic treatment, Finite element analysis, Mineral trioxide aggregate, Calcium enriched mixture, Dental traumatology

## Abstract

**Aim:**

This study aimed to evaluate the stress distribution caused by secondary trauma forces after regenerative endodontic treatment (RET) using different thicknesses of coronary barrier material with three-dimensional finite element analysis(FEA).

**Method:**

A control model was created using the tomography image of the immature maxillary central tooth with computer software.Study models were created with the modulus of elasticity and Poisson’s ratio of the materials used in RET.Enamel, dentin, cementum, periodontal ligament, cortical, and cancellous bone were modeled. Coronary barrier materials were applied in 3 mm and 5 mm thicknesses (Model 1: control model, model 2:3 mm/Calcium Enriched Mixture(CEM), model 3:3 mm/Mineral Trioxide Aggregate(MTA), model 4:3 mm/Biodentin, model 5:5 mm/CEM, model 6:5 mm/MTA, model 7:5 mm/Biodentin). For the trauma force simulation, 300 N force in the horizontal direction was applied to the buccal surface of the tooth in the first scenario. For the second scenario, maximum bite force simulation, a force of 240 N in the oblique direction was applied to the palatal surface of the tooth. FEA was performed with Algor Fempro. The resulting stresses were recorded as Von Mises, maximum, and minimum principal stresses.

**Results:**

Lower stress values were obtained in 5 mm models compared to 3 mm models. However, the difference between them was insignificant. Lower stress values were obtained in all RET models compared to the control model. The lowest stress values in dental tissues and bone tissue were obtained in the CEM models.

**Conclusion:**

This is the first study in which the stress caused by different thicknesses of CEM on dental tissues was evaluated with FEA. RET strengthens immature teeth biomechanically. CEM and Biodentin are more successful materials in stress distribution than MTA. Considering the cost of treatment, 3 mm material thickness is ideal for RET since there is no significant difference between the stress values resulting from the use of 5 mm and 3 mm coronary barrier material.

**Supplementary Information:**

The online version contains supplementary material available at 10.1186/s12903-023-03559-x.

## Introduction

Regenerative endodontic treatment (RET) is a successful option for treating immature teeth. The greatest advantage of RET is that it stimulates immature teeth to complete root development. Increasing the thickness and length of the dentin walls and providing apical closure increase the tooth resistance [1, 2]. For these reasons, RET has recently been preferred by clinicians as an innovative treatment [3].

In contrast to traditional endodontic treatments, there is no root canal instrumentation in RET. The treatment procedure includes canal irrigation and the use of antibiotics, followed by blood clot formation and new tissue regeneration [3]. All stages of the treatment procedure are important for the treatment’s success. One of these steps is to provide a tight coronal plug. Bioactive endodontic cements, such as mineral trioxide aggregate (MTA), calcium enriched mixture (CEM), and Biodentin are used as coronal plugs in RET. Bioactive cements are materials that have proven biocompatibility with dental tissues and are frequently used in endodontics [4]. These materials are applied to the cervical third in RET. According to the literature, one of the shortcomings of RET is the lack of a definitive protocol regarding the ideal application thickness of coronary barrier materials.

MTA is the most preferred material among bioactive endodontic cements [5]. CEM and Biodentin have the properties of MTA; in addition, they also offer the advantages of ease of manipulation, shorter setting time, and color stability [6, 7]. However, they are not used as extensively as MTA due to a lack of evidence-based data in the literature [8].

Maxillary central incisors are the teeth most affected by trauma [9]. The cervical region is especially fragile due to its thin enamel [10]. The teeth must be resistant to the stresses that occur with external forces [11]. After RET, teeth may be exposed to secondary trauma. In this case, the teeth are at risk for fractures due to thin dentin walls [1]. Bioactive cements applied to the cervical third, which is the region where trauma-related fractures occur most frequently in RET, are expected to be compatible with the tooth structure.

It is not ethically appropriate to test the effects of trauma forces in vivo, especially in dentistry [12]. Nor can trauma forces be effectively tested in vitro because supporting tissues with damping effects, such as periodontal ligament and bone tissue, cannot be included in the evaluation [13]. In addition, stress analysis is more informative than fragility tests, as it also determines stress distribution areas [14]. Finite element analysis (FEA) is an engineering method used in biomechanical research. FEA makes it possible to assess stress by including all layers of the tooth and surrounding tissues [15]. For this reason, it is frequently preferred in studies related to biomechanics in dentistry [12].

This study was designed to evaluate the effect of material thickness on stress distribution in the tooth and surrounding tissue as a result of applying horizontal and oblique force to the immature maxillary central tooth with three-dimensional FEA. Except for Part I, which is the predecessor of this study, no study evaluating FEA and CEM has been found in the literature. The current study, Part II, is the first to compare the thickness of CEM as a coronary barrier material in RET.

## Materials and methods

Finite element stress analysis is a non-invasive method used in bioengineering studies. This research is an in vitro, methodological study that utilizes a retrospective tomographic image to evaluate the stress in dental tissues. Ethical approval was obtained for the study (approval number: 2021/1778). For modeling, cone beam computed tomography images (CBCT) with a section thickness of 0.5 mm (taken for diagnostic purposes in the archive of Oral Diagnosis and Radiology of the xxx University Faculty of Dentistry) were scanned retrospectively. All CBCT imaging was performed with the same device (NewTom5G, Quantitative Radiology, Verona, Italy) in the standard supine position (110 kVp, 1–11 mA, 3.6 s). While selecting the image, the immature form, which is the tooth formation in which RET is mostly applied (tooth formation with 2/3 of the root formation completed), was taken into consideration. For this purpose, a healthy, immature maxillary central tooth image of an 8-year-old male patient was selected.

Model 1:(Control Group): Healthy immature maxillary central tooth.

Model 2: CEM—applied tooth as 3 mm coronal plug + glass ionomer cement + composite.

Model 3: MTA—applied tooth as 3 mm coronal plug + glass ionomer cement + composite.

Model 4: Biodentin—applied tooth as 3 mm coronal plug + glass ionomer cement + composite.

Model 5: CEM—applied tooth as 5 mm coronal plug + glass ionomer cement + composite.

Model 6: MTA—applied tooth as 5 mm coronal plug + glass ionomer cement + composite.

Model 7: Biodentin—applied tooth as 5 mm coronal plug + glass ionomer cement + composite.

In models in which RET was simulated, 3 mm and 5 mm thicknesses of MTA, Biodentin, and CEM were applied as coronary plugs. In the models created, the restoration was completed by applying glass ionomer and composite on the coronary barrier material. The modulus of elasticity and poisson ratios of the materials are given in Table 1. These values were used to make a standard evaluation. The number of elements in the models are 540709 in model 1, 535711 in model 2, 535711 in model 3, 535711 in model 4, 538590 in model 5, 538590 in model 6, 538590 in model 7. The The number of nodes are 117,720 in model 1, 114582 in model 2, 114582 in model 3, 114582 in model 4, 115,306 in model 5, 115306 in model 6, 115306 in model 7.

**Table 1:** Elasticity modulus and poisson ratios of dental materials, dental and periodontal structures and the number of elements and nodes of all models

Dental Structure/Material	Modulus of Elasticity (E) (Gpa)	Poisson Ratio	Referance
MTA	11.76	0.31	Aslan 2020
Biodentin	22	0.33	Aslan 2020
CEM	24.87	0.33	^a^
Composite	16.4	0.28	Kuraray America, Tokyo, Japan
Resin modified glass ionomer cement	10.86	0.3	Demirel
Enamel	41	0.31	Aslan 2020
Dentin	18.6	0.31	Aslan 2020
Pulp	0.003	0.45	Aslan 2020
Periodontal ligament	0.0000689	0.45	Aslan 2020
Cancellous bone	1.37	0.30	Aslan 2020
Cortical Bone	13.7	0.30	Aslan 2020
Cement	8.2	0.3	Bucchi-2016

^a^ Nanoindentation test was obtained in the Dokuz Eylul University Engineering Faculty Laboratory

CBCT image volumetric data were exported in Digital Imaging and Communications in Medicine (DICOM) 3.0 format. For analysis, the image in DICOM format was converted to stl format. 3D scanning was used with an Activity 880 optical scanner to create the 3D solid model. VRMesh software was used for the homogeneity of the models and the editing of the 3D mesh structure. The 3D positioning of the models was done with Rhinoceros 4.0 3D modeling software. FEA was performed using Algor Fempro (Algor Inc., USA) software.

**Fig. 1:**
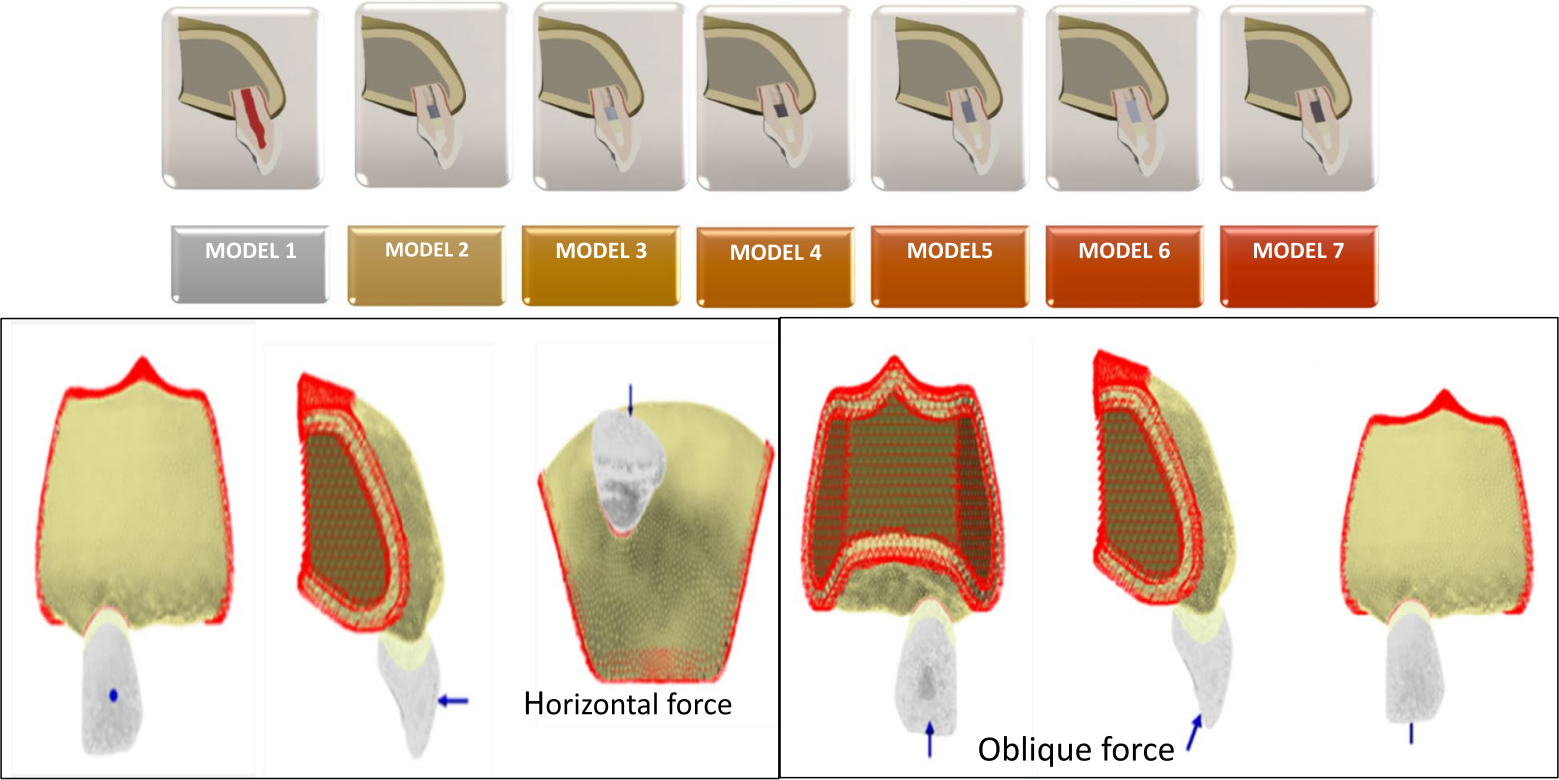
FEA models created for the study and representation of boundary conditions from different views. FEA: Finite Element Analysis

In the models created, enamel, dentin, cementum, periodontal ligament, cortical and cancellous bone were modeled. All the structures of the model were considered homogeneous, linear, and isotropic. Two different forces were applied to the models. For the simulation of the trauma force, a force of 300 N was applied at an angle of 90 degrees in the horizontal direction [16]. For the traumatic bite force simulation, a force of 240 N was applied at an angle of 120 degrees in the oblique direction [17] (Fig. 1).

### Interpretation of analysis results

The stresses occurring in the tooth structures in the models are Von Mises, the maximum and minimum principal stresses, and the stresses occurring in the cortical and cancellous bone are maximum and minimum evaluated as principal stress values. The stress assessment of the materials was recorded as Von Mises stresses. The stress assessment was performed in the cervical area, as it is the most sensitive area to traumas [10]. The stresses occurring in all layers of the tooth were evaluated separately as enamel-dentin-cement-PDL.

Von Mises stress is used to determine the distribution and intensity of stress. Von mises stresses express a scalar stress value. Color scales were used to evaluate the severity of stress. Areas with a color transition from red to blue on the Von Mises color scale indicate the transition from areas of high stress to regions of reduced stress. Principal stresses were used to evaluate the vectorial distribution of stresses. In the basic stress scale, the color scale has positive and negative color representation. Positive values (red, green area) represent tensile stresses, and negative values (blue area) represent compression stresses (Supplementary Figures).

## Results

Stress assessment in each tooth module (enamel, dentin, cementum, periodontal ligament) and bone tissues was performed separately. The color scale of each module was created separately as a result of the values obtained. These color scales are given in Supplemantary figures. Evaluation of the conclusion part was made according to the color transitions obtained from these scales. According to these scales, each force and tooth structure are interpreted separately below.

### Evaluation of stresses in enamel

According to the maximum principal stress scale (A) in Supplementary Fig. 1, the highest stress areas are the areas shown in red. These red areas were formed where the force was applied and in the cervicobuccal region. n the minimum principal stress scale (B), compression type stresses occurred in the blue areas. This region was the cervicopalatinal region. The stress distribution among the models was very similar.

The distribution areas formed by the application of oblique force are visualized in Supplementary Fig. 2. According to the maximum principal stress scale (A) obtained as a result of this distribution; max. Principal stresses (tensile type stresses) occurred in the area where the force was applied and in the cervicopalatinate region. According to the minimum principal stress scale, the blue areas representing the most stress were formed in the cervicobuccal area. The stress distribution between the models was very similar for the two force types.

### Evaluation of stresses in dentin

The stress distribution occurring in dentin with the application of horizontal force is as shown in Supplementary Fig. 3. In the distribution here, according to the maximum principal stress scale (A), the red areas are the places where tensile stress is most intense. Accordingly, red areas representing tensile type stress were found along the buccal root surface. According to the minimum principal stress scale (B), the blue colored areas show intense compression type stress areas. Accordingly, the most intense minimum principal stresses in dentin occurred on the entire palatal root surface. However, the intensity of the color increased significantly in the root dentin. This shows that intense stress areas are concentrated at the root apex.

The stress distribution in dentin caused by the application of oblique force is given in Supplementary Fig. 4. According to the maximum pirincipal scale (A) in this distribution figure, the transition from red areas to blue indicates the transition from high stress areas to decreasing stress areas. Accordingly, stress areas were formed in the root dentin in the palatal region, increasing from the cervical to the apex. The most intense tensile stresses occurred at the apex. According to the minimum principal stress scale (B), areas with a color transition from blue to red indicate the transition from areas of high stress to areas of decreasing stress. Accordingly, there was an increasing stress distribution on the buccal surface of root dentin from cervical to apex.

### Evaluation of stresses in cement

The stress distribution occurring in cement models with the application of horizontal and oblique force is given in Supplementary Fig. 5. According to the maximum principal stress scale (A) in the stress distribution caused by horizontal force, increasing stress areas towards the apex were formed on the buccal surface where the red color is intense. The color change from yellow to red, starting from the cervical and towards the apex, indicates this. With the application of oblique force, blue color, which represents intense stress areas according to the minimum principal stress scale (B), is dominant on the buccal surface.

### Evaluation of stresses in cortical bone

The stress distribution in cortical bone with horizontal force application is given in supplementary Fig. 7. The stress distribution areas in the models were similar. According to the maximum principal stress scale (A), as a result of the horizontal force applied to the cortical bone, compressive stresses occurred in the palatal region and tensile stresses occurred in the buccal/mesial/distal region. As a result of the oblique force, localized intense compression stresses occurred in the buccal and lingual regions.

### Evaluation of stresses in cancellous bone

The stress distribution in the cancellous bone with horizontal and oblique force application is given in supplementary Fig. 8. According to the maximum (A) and minimum (B) principal stress scales in this figure, localized tensile stresses occurred in the buccal and palatal regions as a result of horizontal force, and compressive stresses occurred as a result of oblique force.

**Fig. 2:**
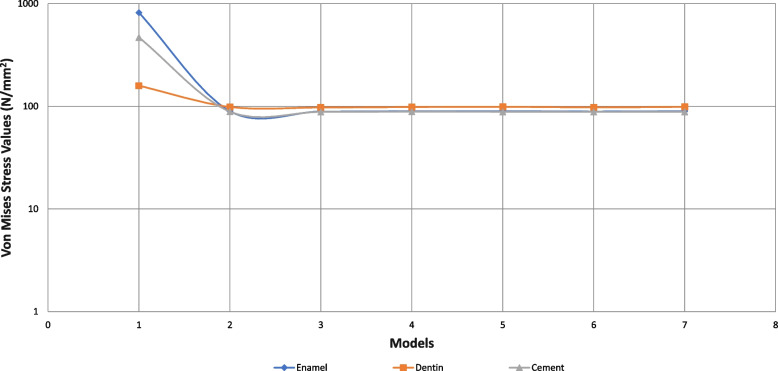
Evaluation of Von Mises Stress Values formed by the application of oblique force with a logarithmic Scale

**Fig. 3:**
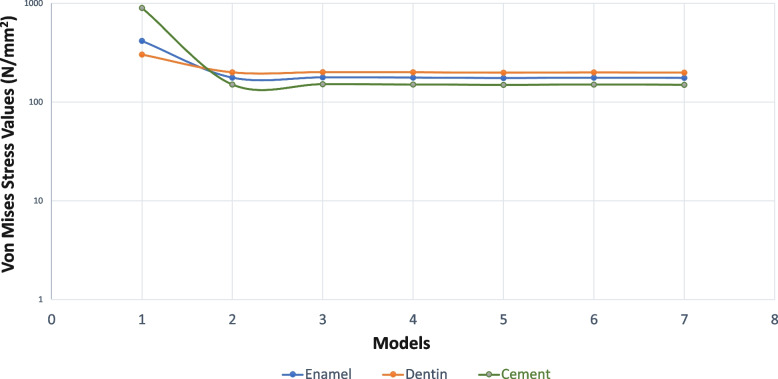
Evaluation of Von Mises Stress Values formed by the application of horizontal force with a logarithmic Scale

Von Mises stress values are given in Table 2. The maximum and minimum principal stresses are given in Table 3. By applying horizontal force to the tooth in enamel, dentin, cementum and PDL, the highest Von Mises stress value was obtained in a healthy, immature tooth (model 1/control) which was not applied RET. Lower Von Mises stress values were obtained in all RET-applied models compared to the control model. Lower stress values occurred in models with 5 mm coronary plugs compared to 3 mm plugs. However, the difference between them was too low to be considered. As a result of oblique force application, much lower stress values were obtained in enamel/dentin/cement/PDL compared to Model 1. The reduction in stress values in cementum was greater than in enamel and dentin. Among the RET models, the least stress in enamel/dentin/cement/PDL was obtained in the CEM/5 mm model (Model 5). The highest stress value occurred in the MTA/3 mm model (Model 3). Among the coronary plugs, the highest Von Mises stress value in the material was obtained in the CEM/5 mm model (Model 5). The lowest stress value occurred in the MTA/3 mm model (Model 3) (Table 2). A comparison of horizontal and oblique force and Von Mises stresses in enamel/dentin/cementum is given in Figs. 2 and 3.

**Table 2:** Von Mises stress values in dental tissues and materials (MPa)

		**Enamel**	**Dentin**	**Cement**	**PDL**	**Composite**	**Glass İonomer Cement**	**Coroner Barier Material**
**Model 1**	**H**	414.0	301.6	894.4	894.4			
	**O**	816.5	158.6	468.9	3.7			
**Model 2 (3mm CEM)**	**H**	175.9	199.1	150.0	6.2	42.4	45.5	160.4
	**O**	89.6	98.5	88.6	3.1	24.4	27.6	80.3
**Model 3 (3mm MTA)**	**H**	177.0	200.1	150.6	6.2	42.5	44.8	83.3
	**O**	88.8	97.4	88.2	3	24.5	27.6	42.1
**Model 4 (3mm Biodentin)**	**H**	176.2	199.3	150.2	6.2	55.3	45.5	144.4
	**O**	89.5	98.4	88.6	3.1	24.4	27.6	72.4
**Model 5 (3mm CEM)**	**H**	174.4	197.4	148.7	6.1	42.2	48.4	194.1
	**O**	89.7	98.8	88.4	3.0	24.3	29.6	127.6
**Model 6 (3mm MTA)**	**H**	175.8	198.8	149.7	6.1	42	47.4	102.1
	**O**	88.9	97.7	88.1	3.1	24	29.3	66.6
**Model 7 (3mm Biodentin)**	**H**	174.7	197.7	148.9	6.1	42.2	48.3	175.1
	**O**	89.6	98.7	88	3.0	24.4	27.6	72.4

*H* horizontal, *O* obligue, *PDL* periodontal ligament

**Table 3:** Maximum and minimum principal values (MPa)

	**F**	**Enamel**		**Dentin**		**Cement**		**PDL**		**Cortical Bone**		**Cancellous Bone**	
		**Max. P**	**Min. P**	**Max. P**	**Min. P**	**Max. P**	**Min. P**	**Max. P**	**Min. P**	**Max. P**	**Min. P**	**Max. P**	**Min. P**
**Model 1**	**H**	73.3	-16.4	145.6	-56.8	292.7	-19.2	5.8	3.5	15.2	-36.3	14.3	-11.6
	**O**	13.3	-46.3	28.0	-90.6	-8.3	-34.6	-1.8	-3.0	10.8	12.7	7.5	5.7
**Model 2**	**H**	42.9	-15.1	101.5	-1.1	102.8	12.2	7.7	4.7	13.4	-34.0	14.0	-11.3
	**O**	12.4	-23.1	0.7	-67.9	-5.5	-62.8	-2.4	-3.9	11.8	12.6	7.4	5.5
**Model 3**	**H**	42.1	-15.5	99.4	-1.1	101.4	11.9	7.7	4.7	13.4	-34	14.0	-11.3
	**O**	12.7	-22.8	0.7	-67.2	-5.4	-62.3	-2.4	-3.9	9.9	12.6	7.4	5.5
**Model 4**	**H**	42.8	-15.1	101.2	-1.1	102.6	12.2	7.7	4.7	13.4	-37.8	14.0	-11.4
	**O**	12.4	-23.1	0.7	-67.8	-5.5	-62.7	-2.4	-3.9	11.8	12.6	7.4	5.5
**Model 5**	**H**	42.7	-14.7	102.0	-1.1	102.9	12.3	7.6	4.6	13.4	-37.8	13.9	-11.3
	**O**	12.2	-23.1	0.7	-68.2	-5.6	-62.9	-2.4	-3.9	11.8	12.0	7.4	5.5
**Model 6**	**H**	42.0	-15.2	99.7	-1.1	101.5	12.0	7.7	4.7	16.0	-37.9	14.0	-11.3
	**O**	12.5	-22.8	0.7	-67.5	-5.4	-62.4	-2.5	-4.1	11.8	12.6	7.4	5.5
**Model 7**	**H**	42.6	-14.8	101.7	-1.1	102.7	12.3	7.9	4.8	15.9	-37.8	13.7	-11.3
	**O**	12.3	-23.0	0.7	-68.1	-5.5	-62.8	-2.4	-3.9	11.8	12.7	7.4	5.5

*H* horizontal, *O* Oblique, *P* principal *F* force

## Discussion

RET has become a frequently preferred treatment method in recent years, especially in necrotized immature teeth [18]. In the current literature, clinical studies evaluating the success of RET and studies examining the factors affecting the success of treatment (irrigation solutions, scaffolds used, appropriate indications) are the majority [19–21]. Coronary barrier materials are also one of the subjects studied by researchers. In the literature, there are studies evaluating the effect of material type on treatment success from various aspects (root length increase/apical diameter narrowing/increase in root thickness) [22]. However, the number of studies evaluating the biomechanical properties of RET-applied teeth is limited. In the literature, biomechanical properties have been evaluated mostly on the basis of fragility [23, 24]. Few studies have evaluated the biomechanical effect of materials on immature teeth by stress distribution [25, 26]. This is the first study in which the effects of using different thicknesses of MTA, CEM, and Biodentin for stress distribution in RET were evaluated together with FEA.

It has been reported in the literature that the application procedure of RET is not clear, and that its deficiencies should be investigated [27]. Knowing the long-term results of studies on RET will enable clinicians to apply this treatment more safely. This study will provide insight into how immature maxillary central tooth tissues and surrounding bone tissue are affected by secondary traumas after RET. In particular, immature teeth are fragile due to their underdeveloped root structures and weak cervical regions [1]. Considering this situation, the effect of coronary barrier materials (MTA/Biodentin/CEM) applied in different thicknesses (3 mm/5 mm) in the cervical region on the stress distribution was evaluated in this study.

The effect of restorative materials on treatment success is important [2]. In the current study, a full RET simulation was performed by including restorative materials (glass ionomer/composite) and all tooth/bone tissues in addition to coronal barrier materials. This situation constitutes the difference between our study and others in the literature.

Biodentin and CEM are successful materials that can be used in many areas where MTA is used. Nevertheless, due to the limited research on the use of these materials in RET, they are less favored than MTA [8]. In this study, In models where CEM and Biodentin were used, the order of stress occurring in tooth and bone tissues was CEM < Biodentin < MTA. In an FEA study evaluating apexification using Biodentin, that material was found to be more successful in stress distribution in immature teeth than MTA similar to the current study[24]. Also in another study conducted with FEA on immature teeth, it was found that Biodentin is more advantageous than MTA [23].

Belli et al. [28] reported that restoration materials reduce the stress on dental tissues. Similarly, the results of the present study revealed that the restoration materials applied in immature teeth reduce and distribute stress. In the same study, it was emphasized that MTA transmits less stress than Biodentin, unlike the results of the current study. A similar result was obtained in an in vitro study evaluating the effects of CEM and MTA on fracture resistance [29]. In our study, MTA transmitted higher stress to dental tissues. While CEM absorbed the most stress, Biodentin showed similar stress absorption to CEM. The lack of complete standardization in in-vitro studies is thought to be the cause of the difference in results.

Bucchi et al. [2] simulated root dentin maturation after RET and compared the stress distribution in immature and mature teeth. In the RET-applied tooth models, the researchers obtained lower stress values than in immature teeth. Similarly, in the results of the current study, lower stress values were obtained in dental tissues and surrounding bone tissue in all RET models compared to the control model. This situation can be interpreted as immature teeth becoming more biomechanically resistant with RET.

In a study in which the effect of MTA thickness on the stress distribution in RET was evaluated with FEA obtained lower stress values in the models in which RET was applied, similar to our study [25]. While Demirel et al. recommend the use of 5 mm material in their study. In the current study there was no significant effect between the 3 mm and 5 mm groups.

If we compare the studies according to the stress distribution areas, in the present study as in the study of Poiate et al. [30] high tensile stresses occurred in the cervical region on the palatal surface of the enamel. The cervical region is also where the enamel is the thinnest, and this area should be supported in treatments for immature teeth [10].

Maximum and minimum stress distribution were observed in the dentin in horizontal and oblique strength, increasing from the cervical region to the apex. This result indicates that stress is concentrated in the apex region of dentin in immature teeth, as in the study of Bucchi et al. [2] Consistent with the results of the study conducted by Antrayoz et al. [31], in which they evaluated the stress caused by oblique force in the maxillary central tooth, the stress level in dentin decreased in all RET models compared to the control model. This result is similar to the results of our study. Stress analysis in models is promising for RET. Lower stress values were also obtained in cementum in the RET models compared to the control. Considering the fine structure of the cementum in immature teeth, the reduction in stress values is a valuable result for the long-term prognosis of immature teeth.

Tensile and compressive stresses are important for dental tissues and materials. In the current study, lower maximum and minimum principal values were obtained in the 5 mm models in enamel, dentin, cementum, and PDL. The decrease in these values is an indication that the resistance of the teeth to the incoming traumatic forces has increased.

Although the stress distribution in cortical and cancellous bone was similar, fewer maximum and minimum principal values were obtained in the RET models. This is an indication of reduced stress in bone tissue. The lowest stress values were obtained in CEM models, as in other dental tissues. This result can constitute an important advantage of the usability of CEM.

Calcium hydroxide, which is used in the second visit in RET, is known to affect the modulus of elasticity of dentin, however, there are studies in the literature that say it reduces [29] fragility and studies that claim the opposite [32]. Since there are no definitive data on this situation, the change in modulus of elasticity in dentin after the use of calcium hydroxide was ignored in the study. In addition, the effects of the solutions used on the dentin tissue were ignored. These situations constitute the limitations of our study.

## Conclusıon

Within the limits of this study, CEM and Biodentin are as successful materials as MTA in terms of biomechanical performance, and the difference between the stress values observed in RET when these materials are applied at 3 mm and 5 mm thickness is too low to be taken into account.

### Supplementary Information

**Additional file 1.**

## Data Availability

The datasets and materials used or analysed during the current study are available from the corresponding author on reasonable request.
