# Supplementary material for: 3D finite element analysis of stress distribution as a result of oblique and horizontal forces after regenerative endodontic treatment part II: comparison of material thickness
Source: BMC Oral Health. 2023 Nov 16;23:869. doi: 10.1186/s12903-023-03559-x (PMC10655308; doi:10.1186/s12903-023-03559-x)
Supplement: Supplementary file 1 — Additional file 1. [file 12903_2023_3559_MOESM1_ESM.zip › Revised Supplementary figure 5.pptx]

## Slide 1
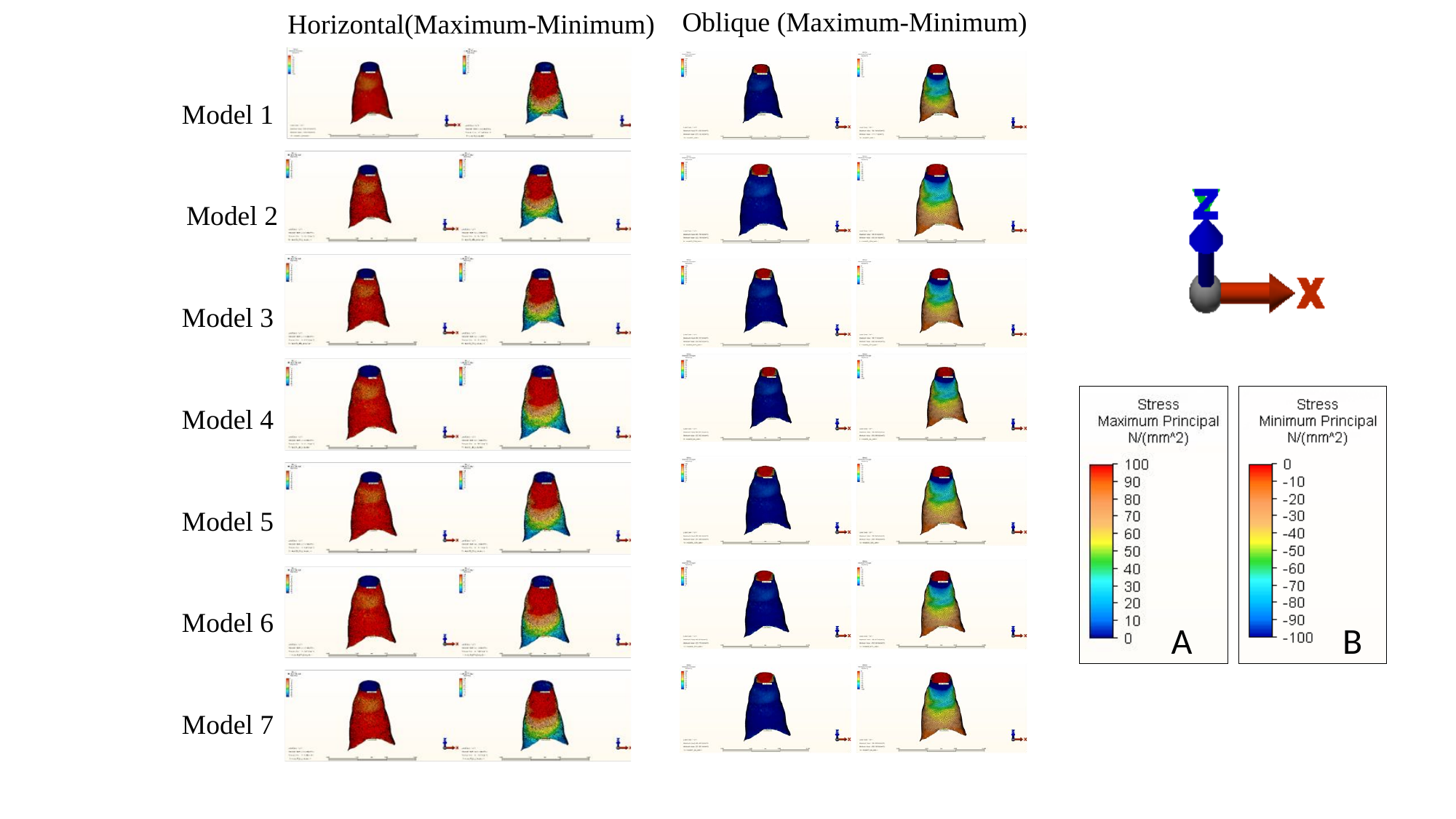

Oblique (Maximum-Minimum)
Horizontal(Maximum-Minimum)
Model 1
A
B
Model 2
Model 3
Model 4
Model 5
Model 6
Model 7
