# Supplementary material for: 3D finite element analysis of stress distribution as a result of oblique and horizontal forces after regenerative endodontic treatment part II: comparison of material thickness
Source: BMC Oral Health. 2023 Nov 16;23:869. doi: 10.1186/s12903-023-03559-x (PMC10655308; doi:10.1186/s12903-023-03559-x)
Supplement: Supplementary file 1 — Additional file 1. [file 12903_2023_3559_MOESM1_ESM.zip › Revised Supplementary Figure Legends.docx]

**Supplementary Figure Legends**

**Supplementary Figure 1.** Maximum and minimum stress distrubution on enamel by applying horizontal force.

Figure description:

Maximum (A) and minimum (B) principal scale, spatial coordinates.Red to blue colors represent stress values from high to low in A. Blue to red colors represent stress values from high to low in B.

**Supplementary Figure 2.** Maximum and minimum stress distrubution on enamel by applying oblique force.

Figure description:

Maximum (A) and minimum (B) principal scale, spatial coordinates. Red to blue colors represent stress values from high to low in A. Blue to red colors represent stress values from high to low in B.

**Supplementary Figure 3.** Maximum and minimum stress distrubution on dentin by applying horizontal force.

Figure description:

Maximum (A) and minimum (B) principal scale, spatial coordinates. Red to blue colors represent stress values from high to low in A. Blue to red colors represent stress values from high to low in B.

**Supplementary Figure 4.** Maximum and minimum stress distrubution on dentin by applying oblique force.

Figure description:

Maximum(A) and minimum(B) principal scale, spatial coordinates. Red to blue colors represent stress values from high to low in A. Blue to red colors represent stress values from high to low in B.

**Supplementary Figure 5.** Maximum and minimum stress distrubution on cement by applying horizontal and oblique force.

Figure description:

Maximum(A) and minimum(B) principal scale, spatial coordinates. Red to blue colors represent stress values from high to low in A. Blue to red colors represent stress values from high to low in B.

**Supplementary Figure 6**. Maximum and minimum stress distrubution on periodontal ligament by applying horizontal and oblique force.

Figure description:

Maximum(A) and minimum(B) principal scale, spatial coordinates. Red to blue colors represent stress values from high to low in A. Blue to red colors represent stress values from high to low in B.

**Supplementary Figure 7.** Maximum and minimum stress distrubution on cortical bone by applying horizontal and oblique force.

Figure description:

Maximum(A) and minimum(B) principal scale, spatial coordinates. Red to blue colors represent stress values from high to low in A. Blue to red colors represent stress values from high to low in B.

**Supplementary Figure 8**. Maximum and minimum stress distrubution on cancellous bone by applying horizontal and oblique force.

Figure description:

Maximum(A) and minimum(B) principal scale, spatial coordinates. Red to blue colors represent stress values from high to low in A. Blue to red colors represent stress values from high to low in B.
